# A genome-based survey of invasive pneumococci in Norway over four decades reveals lineage-specific responses to vaccination

**DOI:** 10.1186/s13073-024-01396-3

**Published:** 2024-10-25

**Authors:** Vegard Eldholm, Magnus N. Osnes, Martha L. Bjørnstad, Daniel Straume, Rebecca A. Gladstone

**Affiliations:** 1 Division of Infection Control, Norwegian Institute of Public Health, Lovisenberggata 6, 0456 Oslo, Norway; 2 Centre for Antimicrobial Resistance, Norwegian Institute of Public Health, Oslo, Norway; 3 Faculty of Chemistry, Biotechnology and Food Science, Norwegian University of Life Sciences, 1430 Ås, Norway; 4 Department of Biostatistics, Faculty of Medicine, University of Oslo, Oslo, Norway

**Keywords:** Streptococcus pneumoniae, Pneumococcal conjugate vaccine, Whole-genome sequencing, Global pneumococcal sequencing clusters, Invasive pneumococcal disease, Serotype replacement, Penicillin resistance, Archival isolates, Population dynamics

## Abstract

**Background:**

*Streptococcus pneumoniae* is a major cause of mortality globally. The introduction of pneumococcal conjugate vaccines (PCVs) has reduced the incidence of the targeted serotypes significantly, but expansion of non-targeted serotypes, serotype replacement, and incomplete vaccine-targeting contribute to pneumococcal disease in the vaccine era. Here, we characterize the changing population genetic landscape of *S. pneumoniae* in Norway over a 41-year period (1982–2022).

**Methods:**

Since 2018, all cases of invasive pneumococcal disease have undergone whole-genome sequencing (WGS) at the Norwegian Institute of Public Health. In order to characterize the changing population over time, historical isolates were re-cultured and sequenced, resulting in a historical WGS dataset. Isolates were assigned to global pneumococcal sequence clusters (GPSCs) using PathogenWatch and assigned to serotypes using in silico (SeroBA) and in vitro methods (Quellung reaction). Temporal phylogenetic analyses were performed on GPSCs of particular interest.

**Results:**

The availability of WGS data allowed us to study capsular variation at the level of individual lineages. We detect highly divergent fates for different GPSCs following the introduction of PCVs. For two out of eight major GPSCs, we identified multiple instances of serotype switching from vaccine types to non-vaccine types. Dating analyses suggest that most instances of serotype switching predated the introduction of PCVs, but expansion occurred after their introduction. Furthermore, selection for penicillin non-susceptibility was not a driving force for the changing serotype distribution within the GPSCs over time.

**Conclusions:**

PCVs have been major shapers of the Norwegian disease-causing pneumococcal population, both at the level of serotype distributions and the underlying lineage dynamics. Overall, the introduction of PCVs has reduced the incidence of invasive disease. However, some GPSCs initially dominated by vaccine types escaped the effect of vaccination through expansion of non-vaccine serotypes. Close monitoring of circulating lineages and serotypes will be key for ensuring optimal vaccination coverage going forward.

**Supplementary Information:**

The online version contains supplementary material available at 10.1186/s13073-024-01396-3.

## Background

*Streptococcus pneumoniae* remains a major cause of mortality and death globally. In Norway, the incidence of invasive pneumococcal disease (IPD) has decreased over the last two decades, in parallel with the introduction of childhood vaccination [1]. Specifically, the introduction of the 7-valent conjugate vaccine (PCV7) in 2006 and the 13-valent vaccine (PCV13) in 2013 has been shown to reduce the incidence of the targeted serotypes significantly [2]. In 2022, both extended valency vaccines PCV15 and PCV20 were approved for use by the European Medicines Agency in those > 6 weeks old and > 18 years old respectively for the continued control of pneumococcal disease [3, 4]. Up-to-date intel on circulating serotypes, and their contribution to IPD, will thus be key for choosing the most appropriate vaccines for vaccination programs going forward.

The vaccines are based on capsular polysaccharides of a selection of pneumococcal serotypes. Capsule diversity is however substantial in pneumococcal populations. The polysaccharide composition of the pneumococcal capsule is determined by the *cps* locus, which is delimited by *aliA* and *dexB*, and hosts a varying number of genes involved in capsule synthesis [5, 6]. Capsule variation is mainly driven by genetic recombination between pneumococcal strains, creating the opportunity for “serotype switching” [7]. The incorporation of very long DNA fragments into the recipient genome often leads to the acquisition of an entirely new capsule type or, for nonencapsulated clones, the de novo acquisition of a functional capsule [8], whereas the acquisition of shorter fragments often results in new, but related, capsule types within the same serogroup [7, 9]. Studies from around the world have shown that the reduction of IPD brought about by the use of PCVs has been offset by the increase in IPD due to serotypes not contained in the vaccine [10]. Such replacement can be mediated by two mechanisms: (i) the expansion of non-vaccine type (NVT) lineages and (ii) the expansion of NVTs within a lineage previously dominated by vaccine types (VTs). The latter was shown to be the dominant mechanism in an international genomic comparison of pre and post-PCV invasive pneumococcal disease cases [11].

In addition to NVT disease, a better understanding of residual VT infections is needed. The latter could reflect insufficient real-world efficacy, as has been seen for serotype 3 [12], unrecognized serotypes within a serogroup [13] for which cross-reactivity may be insufficient to reduce disease cases, or possibly regulation of cps gene expression. More generally, residual VT disease could be the result of insufficient herd protection or, more concerning, vaccine breakthroughs and failures [14]. The reasons for residual VT disease are not fully understood.

The exact lineages involved in replacement and VT evasion are a function of the pre-PCV population structure [15–17] making it important to consider the pneumococcal population history within a country to understand post-PCV disease.

Whole genome sequencing (WGS) has rapidly evolved into the most powerful tool for molecular epidemiological surveillance. A number of key metrics, including penicillin susceptibility [18], serotype [19], and genome-level strain clusters [20], can be obtained from sequence data alone, and comprehensive databases exist for contextualization and surveillance (see, e.g., [20, 21]). Whole-genome sequencing was introduced as the main tool for surveillance of IPD in Norway in 2018. In order to conduct a WGS-based longitudinal study of pneumococci circulating in Norway, we additionally re-cultured and sequenced clinical pneumococcal isolates collected between 1982 and 2018. This allowed us to characterize the changing population genetic landscape of *S. pneumoniae* over a 41-year period (1982–2022). Lineages were assigned employing the Global Pneumococcal Sequence Cluster (GPSC) framework [17, 20], which defines lineages based on whole-genome kmer-clustering against a curated and regularly updated database.

This genomic dataset, spanning four decades, provides an opportunity to contextualize post-PCV disease trends in Norway. Here, we investigated the changing prevalence of lineages (GPSCs) and serotypes in relation to the introduction of PCV7 and PCV13. Serotype switching, resulting in vaccine escape, has been a concern after the introduction of PCVs. We found large-scale serotype replacement to have occurred in two of the most frequently observed GPSCs, namely GPSC6 and GPSC7, and performed phylogenetic analyses to better understand their evolution in response to vaccination. Finally, we assessed the potential vaccine coverage of lineage-NVT combinations that have significantly increased in incidence post-PCV13.

## Methods

### Culturing, DNA extraction, and sub-sampling of isolates

For the years predating routine genomic surveillance, we aimed to sequence 40 isolates per year, but we were unable to retrieve samples from some years (1991–1994 and 2011) due to missing or misplaced records. For some years, failed culturing also contributed to < 40 WGS results being available. For all years for which isolates could be retrieved, we included all isolates with a measured penicillin G MIC ≥ 4 μg/ml, whereas the remainder of the isolates were selected at random. For the period covered by routine genomic surveillance, the same logic was applied. Isolates were grown on Columbia agar containing 5% defibrinated horse blood. DNA was extracted using the QIAamp® DNA Mini kit (QIAGEN) on the Qiacube platform following the manufacturer’s protocol. The following pretreatment was employed for improved lysis: a full smear loop of bacterial growth was added to an Eppendorf tube containing 100 μl ATL, 20 μl proteinase K and 4 μl RNase A, vortexed, and incubated at 56 °C for 1 h with shaking at 450 rpm. Sequencing was performed on the Illumina NextSeq and MiSeq platforms, and all WGS data is available via the Global Pneumococcal Sequencing Project Monocle data viewer [22].

Two overlapping sequence collections were used for different analyses. The “historical dataset” (1982–2022), which aimed to include approximately similar number of genomes from all years, included a total of 1305 genomes, of which 1243 were randomly selected and 62 were included on the basis of penicillin resistance (MIC > 2 μg/ml). The “Full dataset” (*n* = 2424) included the same 1305 genomes but additionally included all genomes sequenced in the period of routine genomic surveillance (2018 and forward). In the historic dataset, isolates sampled in the years 2018–2022 comprised 201/1305 (15.4%) of the genomes, compared to 1320/2424 (54.5%) of the genomes in the full dataset. The Norwegian Institute of Public Health (NIPH) receives cultures from invasive pneumococcal infections, as IPD is notifiable. Among the 1305 isolates in the historical dataset, 364 were from blood culture, 12 from cerebrospinal fluid, and 929 of unknown source. The large fraction of unknowns is related to incomplete records for older samples. For the full dataset, enriched for more recent samples, 1275 were from blood culture, 44 from cerebrospinal fluid, and 999 of unknown source. See Additional file 2 for a description of the full dataset.

### Sequence analyses and annotations

Sequencing reads were generated on the Illumina MiSeq and NextSeq platforms and end-trimmed using Trimmomatic [23]. Genomes were subsequently assembled using Spades v3.15.0 [24] in “careful” mode. Capsule serotyping was done by Quellung reaction using serotype-specific sera (isolates collected before 2018) or in silico using SeroBA v1.0 [19]. Isolates assigned to serogroup 24 by SeroBA were further serotyped by the Quellung reaction. Assemblies were assigned to Global Pneumococcal Sequence Clusters (GPSCs) using PathogenWatch (www.pathogen.watch), which uses PopPUNK [25] to assign isolates based on a pre-defined database [17], as described on the GPS Tools site [26].

### Statistical analyses

Epidemiological data were analyzed and visualized in R, using basic functionalities in addition to the packages *dplyr* [27], *ggplot2* [28], *tidyverse* [29], and *RColorBrewer* [30]. Incidence rate ratios (IRR) for GPSCs were modeled in R using a previously published approach [11]. Briefly, we estimated the IPD cases from the observed genomic counts from the full dataset (*n* = 2424 without subsampling down to ~ 40 per year) by adjusting by the proportion of total cases sequenced in each year. We then used regression to calculate the IRRs for the VT and NVT components of GPSCs that allowed the temporal trends within the pre-PCV (1984–2005) and post-PCV13 (2011–2022) periods to be utilized. If overdispersion was detected in the initial Poisson regression, we assessed for a minor violation (goodness-of-fit GOF, *p*-value > 0·01 and < 0·05) or used the negative binomial regression (Poisson GOF *p* value < 0·01). Any zero inflation was reported. If neither model fit, we calculated IRRs using the average annual incidence for the two periods. If a GPSC was not observed in one of the two PCV periods, we added a constant number of 1 to the estimated IPD cases to avoid IRRs being calculated as zero or infinity.

### Temporal phylogenetic analyses

For GPSC-specific analyses, the oldest isolate within each group was used as reference (GPSC6: GPS_NO_NIPH_115, GPSC7: GPS_NO_NIPH_1402) and annotated with Bakta v1.8.2 [31] using the Bakta light database. Sequencing reads from isolates assigned to GPSC6 and GPSC7 were aligned to their GPSC-specific reference using Snippy [32] with a minimum coverage of 8 and a minimum fraction of 0.9 required to call a base. The Snippy core module used to generate genome-wide alignments.

The genome-wide alignments generated by Snippy core was used as input for recombination detection using Gubbins v.3.3.1 [33] with IQ-TREE [34] for tree-building ( –tree-builder iqtree –sh-test –best-model –extensive-search –iterations 8). The Gubbins output was used to generate dated trees with BactDating v.1.1.2 [35] with additive uncorrelated relaxed clock (arc) and useRec = T, with 10 million steps, and keeping 1000. The first 500 trees were removed as burn-in and the maximum clade probability tree selected using TreeAnnotator [36]. To assess the temporal signal in the GPSC-specific datasets, the sampling dates were randomized 10 times for each dataset, and the mutation rate was estimated independently using BactDating as above. The estimated mutation rate ranges (95% confidence intervals) did not overlap with the estimates using the real sampling dates, confirming that a temporal signal was present in both datasets. The estimated rates for GPSC6 were 0.25 substitutions/genome/year outside of identified recombination tracts (95% CI: 0.20–0.30), whereas the corresponding estimates for GPSC7 were 0.40 (95% CI: 0.29–0.53). Root-to-tip plots are included in Fig. S1. Detected recombinations were visualized together with the dated trees using phandango [37], and final figures were post-processed with Adobe Illustrator.

## Results

### IPD incidence over time

**Fig. 1:**
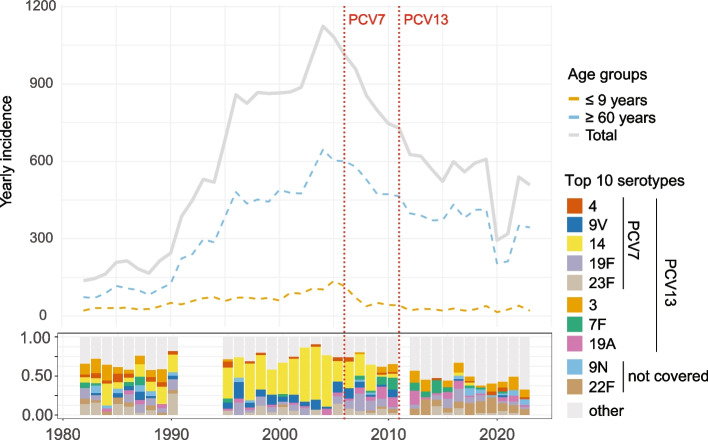
Incidence of invasive pneumococcal disease by age group. The top panel shows incidence data from the Norwegian Surveillance System for Communicable Diseases (MSIS) by age groups. The bottom panel shows the distribution over time of the ten most abundant serotypes from a historical genome dataset (see text for details). Serotypes covered by the vaccines PCV7 and PCV13 are annotated. The “other” group includes 42 different serotypes as well as non-typable isolates

In order to visualize the yearly incidence of invasive pneumococcal disease in Norway over the last four decades, data was extracted from the Norwegian Surveillance System for Communicable Diseases [38]. Following a long period of sustained increase in incidence, the trend reversed from 2004. PCV7 became available in 2001 and was added to the childhood vaccination program in 2006. In the youngest age group, the introduction of PCV7 was followed by a substantial decrease in IPD incidence in the following years (Fig. 1).

In order to broaden the protective effect of vaccination, PCV13 replaced PCV7 in the childhood vaccination program in 2011, which entailed an expansion of the original seven covered serotypes with an additional six.

Since 2018, all pneumococcal isolates shipped to the Norwegian Institute of Public Health (NIPH), of which the vast majority represent invasive cases, as IPD is mandatory notifiable, have undergone whole genome sequencing for genome-epidemiological purposes. However, the pneumococcal isolate collection at NIPH stretches back to the early 1980s, and we wanted to take advantage of this collection to shed light on the historic population dynamics of pneumococci. We particularly aimed to characterize how the pneumococcal population has evolved in response to the addition of pneumococcal conjugate vaccines to the childhood vaccination program. We generated a historical genome dataset spanning 41 years by combining sequence data from re-cultured historical isolates with genomes generated as part of routine surveillance since 2018. In total, this historical collection consisted of 1243 genomes randomly subsampled to reflect the real IPD burden over time, and 62 genomes included on the basis of exhibiting penicillin G MIC above the non-meningitis MIC threshold of MIC > 2 μg/ml (see methods), for a total of 1305 genomes. We also affirmed that the age-distribution of sequenced cases over time in the historical dataset (Fig. S2) reflected the trends in the surveillance data (Fig. 1), that is, a relatively stable age-distribution through time, apart from a reduction of cases among young children following the introduction of PCVs in the childhood vaccination program.

**Fig. 2:**
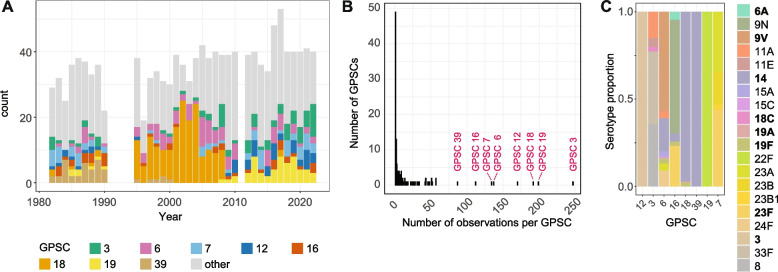
Top GPSCs in Norway over four decades. **A** Yearly frequency of GPSCs in the historic (subsampled) dataset. **B** Total number of isolates by GPSC including all isolates, that is, recent years not subsampled. The eight largest GPSCs are annotated. **C** Serotype distribution within the eight largest GPSCs. Serotypes covered by PCV13 are indicated by bold letters

The GPSC distribution per MIC category is illustrated in Fig. S3. The number of isolates included per year, highlighting the eight most frequent GPSCs, is visualized in Fig. 2.

### Diverging fates of lineages in response to vaccination

**Fig. 3:**
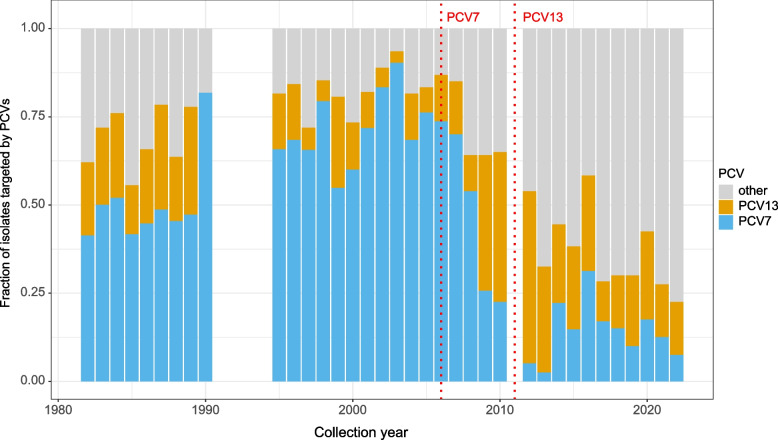
Fraction of isolates carrying PCV7 and PCV13 serotypes over time. PCV13 serotypes include the PCV7 serotypes in addition to six unique serotypes. The annotated dotted lines indicate the year of introduction of the vaccine in the childhood vaccination program

Next, we investigated serotype distribution over time, specifically, the fraction of isolates in each year that had capsule types covered by the PCV7 and PCV13 vaccines (Fig. 3). We observed a clear reduction in PCV7-covered serotypes among IPD cases following the introduction of the vaccine in 2006, in line with earlier observations of reduced carriage [39]. Compared to PCV7, the effect of PCV13 vaccination was more moderate, but there is still a clear decrease in the fraction of all IPD cases made up of PCV13 serotypes, following the introduction of PCV7 and PCV13.

Understanding pneumococcal capsule variation is of major importance for the design and implementation of vaccine programs. However, the serotypes of individual strains say little about the underlying genetic relationships between them, as serotypes can be replaced through recombination [7]. Thus, in order to better understand capsule variation and selection within lineages, we investigated the fraction of isolates covered by PCV7 and PCV13 at the level of individual GPSCs.

**Fig. 4:**
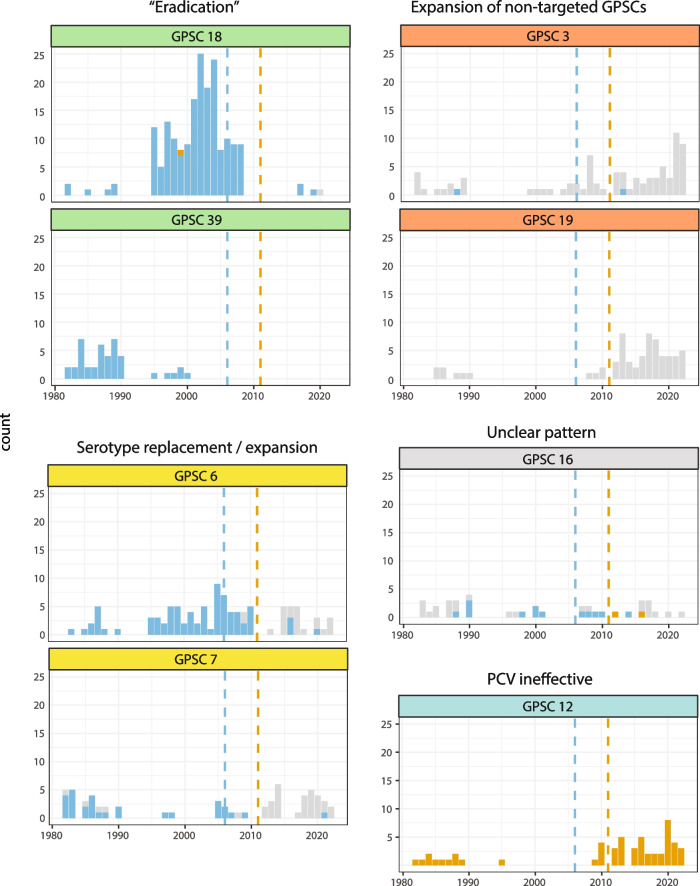
Distribution of PCV-targeted serotypes over time within major Norwegian GPSCs. The dotted vertical lines represent the year of introduction of the PCV7 (blue) and PCV13 (orange) vaccines. Serotypes covered by the PCV7 vaccine in blue; serotypes covered by PCV13 only (i.e., the six serotypes unique to PCV13) in orange. Serotypes not covered by either in grey. The GPSCs have been sorted by different groups by PCV coverage and response to introduction of PCVs

The eight most commonly observed GPSCs, accounting for a total of 48% (627/1305) of the subsampled dataset, were selected for individual analyses (Fig. 2). We plotted yearly counts of each GPSC, coloring each observation by whether the isolate carried a capsule covered by PCV7 and/or PCV13 (Fig. 4). We also estimated incidence rate ratios (IRRs) for each GPSC to detect significant changes in VT or NVT incidence between the pre-PCV (< 2006) and post-PCV13 (> 2011) periods. At the level of individual GPSCs, we observed a number of different fates and responses to the introduction of PCVs.

GPSC18 and GPSC39, which are almost completely dominated by PCV7 serotypes, have been nearly eradicated following the introduction of PCVs (IRR 0.01, *p* < 0.0001, IRR 0.04, *p* < 0.0001 respectively). GPSC16 was made up of a mix of vaccine types and non-vaccine types both before and after vaccination, but only non-vaccine types have been observed since 2017. The increase in the incidence of GPSC16-NVT post-PCV13 (predominantly 9N in adults) was not significant. Non-vaccine type capsules dominated post-PCV13 in GPSC19 (22F 100%) and GPSC3 (8 70%, 33F 15% 11A 10%, 11E 3%), both of which have expanded rapidly post-PCV13 (NVT IRR 22.0, *p* < 0.0001, NVT IRR 3.6 *p* = 0.02, respectively). Together, these lineages account for 33% (92/278) of NVT disease post-PCV13 (Fig. 4). GPSC12 stands out, with 169 out of 170 isolates belonging to serotype 3, covered by PCV13; this lineage has increased in incidence post-PCV13 (VT IRR 9.6, *p* = 0.0001) and accounted for 22% (30/134) of residual VT disease post-PCV13. To investigate whether the failure of PCV13 to reduce the incidence of GPSC12 could be related to the age distribution within the GPSC, we compared it to the age distribution within GPSC18 and GPSC39, which were successfully curbed by PCV7. We found that the age distribution of GPSC12 and GPSC18 cases were not significantly different, suggesting that other factors were involved in shaping their response to vaccination (Fig. S4). This observation adds to a large number of reports suggesting that PCV13 offers limited protection against serotype 3 pneumococcal infection [40–44].

Finally, of particular interest, GPSC6 and GPSC7 exhibited clear signatures of serotype replacement, with a transition from vaccine types dominating in the pre-vaccine era (VT IRR GPSC6 0.05 *p* < 0.0001, GPSC7 0.01 *p* = 0.0003) and non-vaccine types dominating after the introduction of PCVs (NVT IRR GPSC6 19.1 *p* < 0.0001, GPSC7 23.3 *p* < 0.0001, Fig. 4).

A further 9 lineages had increasing NVTs (NVT IRR > 1, *p* < 0.05, Table S1). Most lineages with expanding NVTs (12/13) contain at least one NVT that is also not included in the recently approved extended valency vaccines (PCV15 and PCV20) and four of those 12 are contain NVTs not found in PPSV23 or the experimental PCV21 (Merck V116). GPSC9, GPSC47, and GPSC72 are additionally concerning as they have a higher proportion of NVTs that are penicillin non-susceptible for the EUCAST v14.0 meningitis break-point of ≥ 0.06 μg/ml (100%, 100%, 30% respectively, Table S1). Only 6.2% of these expanding GPSC-NVTs were from children ≤ 9 years old, 65% were in those ≥ 60, and the remaining 29.0% were in those 10–59 years.

### Serotype switching generally predated vaccination

**Fig. 5:**
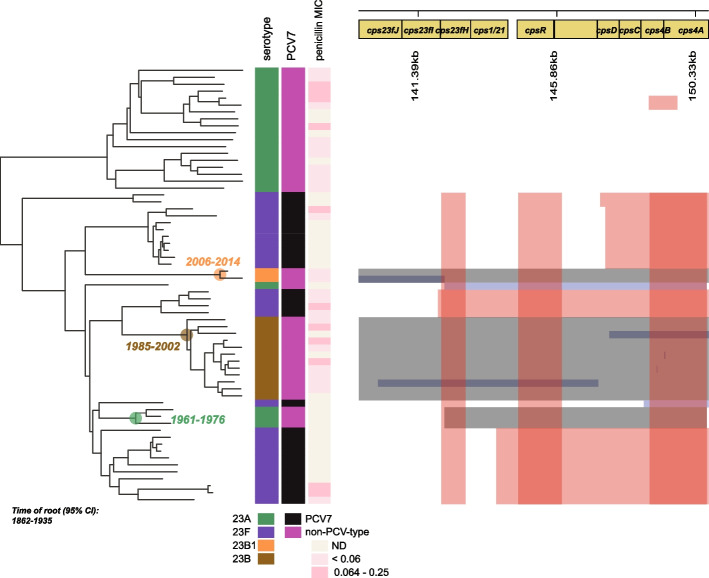
GPSC7 serotype switching. Three events of switching from 23F to other capsule types are highlighted. Identified recombination events in the capsule locus to the right. Events that could be linked to serotype-switching from vaccine to non-vaccine types are colored in gray

Serotype-switching has been highlighted as a potentially worrisome response to vaccines targeting the pneumococcal capsule, and we therefore decided to study GPSC6 and GPSC7, exhibiting a clear history of serotype-switching (Fig. 4), in more detail. We first generated recombination-corrected trees using Gubbins [33] and subsequently generated dated trees from the Gubbins output using BactDating [35]. Serotype-switching events were defined as instances where a monophyletic clade had acquired a new trait (serotype) relative to its closest neighbor. For GPSC7, three serotype-switching events from PCV7 serotypes to non-vaccine types were identified (Fig. 5). The nodes representing the most recent common ancestors (MRCAs) for these clades (highlighted in the figure), represent the last possible time of switching, as the switching events could have occurred anywhere on the branch leading to these nodes. Still, two out of three events, acquisition of serotype 23B (node age 95% CI: 1985–2002) and 23A (node age 95% CI: 1961–1976), clearly predated the introduction of PCV7 in 2006, whereas a third, acquisition of serotype 23B (node age 95% CI: 2006–2014), could have occurred before or after PCV7 introduction. However, there is a single genome of the same ST(1448) on pubMLST isolated in the USA in 1998, which we confirmed is also genotype 23B1, indicating that the switch likely occurred pre-PCV7.

**Fig. 6:**
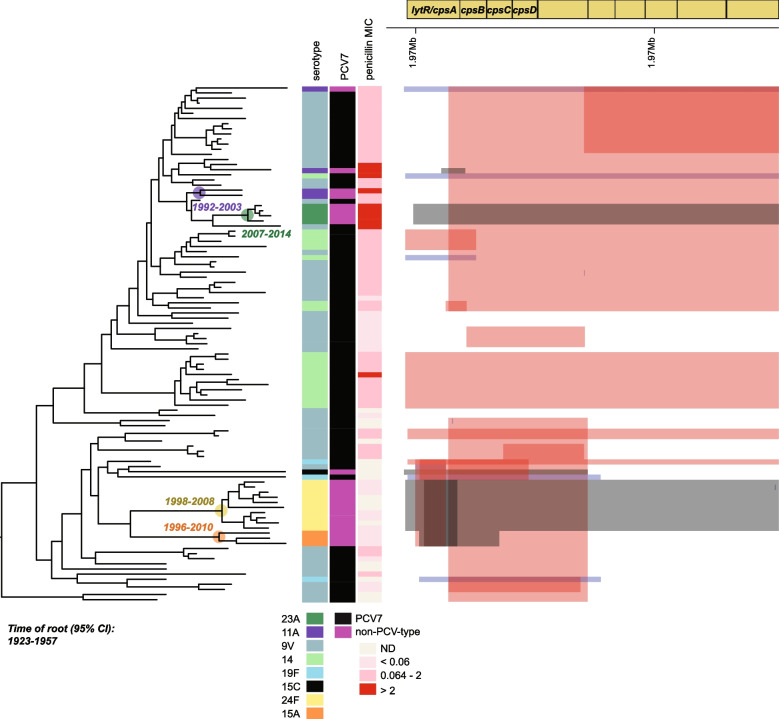
GPSC6 serotype switching. Switching-events from vaccine type to non-vaccine types are highlighted. Identified recombination events in the capsule locus to the right. Events that could be linked to serotype-switching from vaccine to non-vaccine types are colored in gray

For GPSC6, the pattern was found to be similar, a single switching event from vaccine type to non-vaccine type, namely the acquisition of serotype 23A (node age 95% CI: 2007–2014) could have occurred before or after the introduction of PCV7. While the date of the most recent common ancestor is post-PCV7, the acquisition event could have occurred anywhere on the branch leading to this cluster. There are no instances of 23A single locus variants (SLVs) of the ST9946 in pubMLST or PathogenWatch isolated pre-PCV, the earliest being from 2013 in Hong Kong, so we cannot rule out that the switch in fact occurred post-PCV. However, three other events, acquisition of 11A (node age 95% CI: 1992–2003), 15A (node age 95% CI: 1996–2010), and 24F (node age 95% CI: 1998–2008), most likely predated PCV7 introduction (Fig. 6). There was one 11A SLV of ST166 in pubMLST isolated in 1997 from South Korea in line with our estimate of the date of acquisition, but no pre-PCV SLVs of 15A-ST3811 or 24F-ST162 were observed in pubMLST or PathogenWatch that would have proved they existed pre-PCV.

### Selection for penicillin resistance is not a driver of serotype distribution

**Fig. 7:**
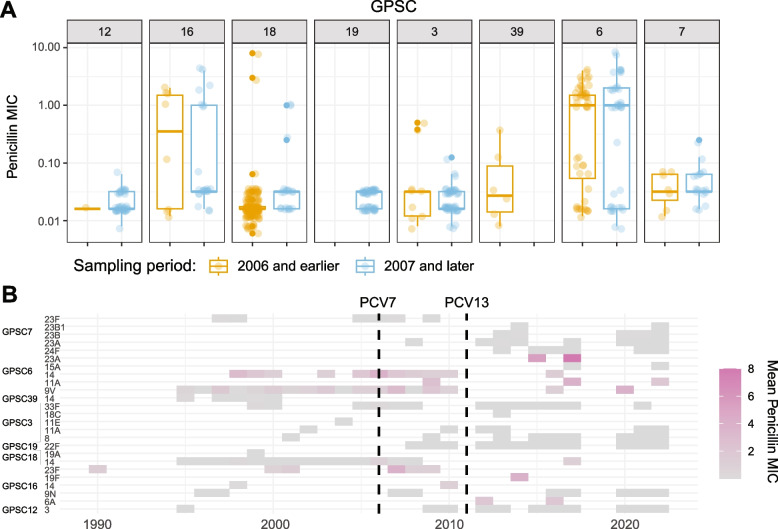
Penicillin MICs stratified by GPSCs and serotypes. **A** Penicillin MICs within top eight GPSCs, stratified by sampling period (before and after the introduction of conjugate vaccines). **B** Mean yearly penicillin MICs by serotype within major GPSCs

As penicillin is the preferred antibiotic for the treatment of pneumococcal infections, and as increasing proportions penicillin non-susceptible NVTs have been reported over time [11], we investigated whether changes in serotype distributions within GPSCs could be related to penicillin MICs. It is conceivable, if particular serotypes co-segregate with alleles conferring reduced penicillin susceptibility, that selection for resistance could play a role in determining observed serotype distributions. We found that penicillin MIC distributions did indeed differ between serotypes within individual GPSCs but found no evidence for penicillin susceptibility influencing serotype selection. Within the most common GPSCs, MIC distributions were similar before and after the introduction of PCVs (Fig. 7A), despite observed changes in serotype distribution (Figs. 4 and 7). As the dataset was biased towards inclusion of penicillin resistant isolates, we did however not perform statistical trend analyses.

## Discussion

In addition to reducing the incidence of the targeted serotypes, it has been convincingly shown that PCVs have significantly reduced childhood pneumococcal carriage in general [2] as well as IPD incidence [45, 46]. In parallel, however, increasing incidence of non-PCV13 serotypes has dampened the effect of childhood PCV13 vaccination programs [45]. The expansion of non-vaccine types can be driven either by the expansion of circulating NVTs or by serotype-switching, i.e., a recombination event in the cps locus within a lineage replacing their vaccine-targeted capsules by non-vaccine types after PCV introductions [7, 47].

The genetics of the pneumococcal capsule and its synthesis is well understood [6, 48]. Furthermore, the sequencing and characterization of historical pneumococcal genomes has added substantially to our knowledge of capsular diversity [49]. Here, by sequencing the genomes of historical Norwegian pneumococcal isolates, we were able to characterize country-level pneumococcal lineage dynamics over four decades. Importantly, the availability of genome sequences allowed us to study capsular variation at the level of individual lineages, specifically GPSCs [17]. This is a key strength of the current study, as without sequence data, population-level analyses cannot shed light on the population dynamics underlying changes in serotype distributions.

Analyses of serotype diversity over time revealed highly divergent outcomes for different GPSCs and serotypes, in response to the introduction of PCVs. Among the most commonly observed GPSCs in Norway over the last 40 years, GPSC18 and GPSC39, almost exclusively harboring PCV7-targeted serotypes, were practically eradicated following the introduction of PCV7. GPSC18-16F and 7C have been observed causing disease in both North and South America post-PCV and GPSC18-24F caused post-PCV13 cases in France, but they are not a significant cause of post-PCV NVT disease [50, 51]. One hypothesis for why some NVT-lineage combinations expand more than others, is negative-frequency dependent selection [15], the phenomenon that accessory genes have an equilibrium frequency at the population level. Specifically, this means that rare genes are generally advantageous, until reaching a certain frequency, at which point they are no longer favored. This, in turn, influences the success of lineages depending on their accessory gene content. There is also strong population structure in the pneumococcus, both in that isolates from the same country often share a common ancestor within clones and that the different countries have different combinations of lineages that are present, given the history of pneumococcal migration. The expansion of specific lineages is thus also often country-specific. For example, it was recently estimated that the migration of strains between countries in Africa can take at least four decades before that strain is equally likely to be found in another African country as in the original country [52]. Problematic GPSC-serotype combinations are more likely to be imported from neighboring countries or those with high connectivity, though this may still occur in the timeframe of decades [53]. Conversely, the already globally disseminated GPSC12 that is completely dominated by serotype 3 isolates targeted by PCV13 was found to have expanded in Norway after the introduction of PCV13, as in the UK and US [41, 54]. This counterintuitive finding mirrors earlier studies demonstrating that PCV13 fails to effectively target this serotype [40–42]. More in line with prior expectations, GPSC3 and GPSC19, completely dominated by non-vaccine serotypes, were found to have expanded in Norway in the wake of PCV7 and PCV13 introduction, as was observed for GPSC3 in South Africa [11]. PCV15, PCV20, and the existing 23-valent Pneumococcal Polysaccharide Vaccine (PPV23) all include serotype 22F which would protect against all GPSC19 and 33F found in GPSC3. PCV20 and PPV23 additionally include 8 and 11A covering the bulk of the expanding GPSC3; however, it is unclear what the extent of cross-protection against 11E will be. 11E was the only NVT serotype observed in GPSC3 that is not in any extended valency PCV (licensed or experimental) or PPV23.

GPSC6 and GPSC7, initially dominated by vaccine type capsules, were mainly observed as non-vaccine types after the introduction of the vaccines. Relying on temporal evolutionary analyses, we were able to date individual serotype-switching events. The analyses revealed that the acquisition of 11A, 15A 24F, 23A, and 23B generally predated the introduction of the vaccines. In other words, in the case of GPSC6 and GPSC7, substantial capsule diversity already present at the time of PCV-introduction, allowed the introduced selective pressure to favor non-vaccine types. Unlike observations from the UK [55], GPSC6 was not a significant cause of post-PCV13 cases of 19F in Norway; in fact, this combination was only seen 4 times across the entire collection. Lineages prevailing post PCV7/13 contain multiple serotypes that are not included in PCV15 and PCV20 but would be covered by the experimental PCV21 (Merck V116). Genomic data has demonstrated the long history of serotype switching events that have occurred in the pneumococcal population, with pneumococcal lineages expressing a diverse set of serotypes [17, 51]. Hence, our finding that PCV-induced selection predominantly resulted in the expansion of pre-existing NVTs, rather than contemporaneous switch events, should not come as a surprise. Yet, serotype switching has been a common occurrence in pneumococcal history and can be expected to continue to occur. Going forward, the early detection of locally expanding NVT-lineages will be important, especially those that are not in planned extended valency PCVs. Further expansion of GPSC47-serotype 6C could become an issue with penicillin MICs of 0.125–0.25 which are above the EUCAST v14 meningitis breakpoint of ≥ 0.06 μg/ml. Though GPSC6 11A and 23A are the only NVTs found in NVT-GPSC expansions for which some MICs are above the non-meningitis cutoff of > 2 μg/ml (11A 2–4 μg/ml, 23A 4–8 μg/ml), these serotypes would be covered by the experimental PCV21 (Merck V116). When looking at the NVT serotypes found in these expanding GPSC-NVTs, PCV15, PCV20, and PPV23 cover two of NVTs reported to be highly invasive (22F, 8) but only PCV21 (merck V116) would cover 24F [17, 56]; the remaining serotypes involved in NVT-GPSC expansions are not highly invasive and may represent large expansions and spillover from asymptomatic carriage to vulnerable populations. In Sweden, GPSC9 was shown to be responsible for 5/25 cases of multidrug-resistant IPD in 2016–2018 [57], whereas in our Norwegian dataset, all 33 GPSC9 isolates exhibited penicillin MICs above the meningitis-breakpoint, but none exhibited MICs > 2 μg/ml.

There was no indication in our data that selection for decreased penicillin susceptibility was a driver of the observed changes in capsule types over time within major GPSCs in Norway. The five most common GPCSs in the last decade (2013–2022) were GPSC19, GPSC3, GPSC12, GPSC7, and GPSC6. Among these, all except GPSC6 were clearly dominated by penicillin-susceptible isolates (Fig. 7). GPSC6 is also interesting, as all isolates carrying a 24F capsule exhibited penicillin susceptibility. The 24F serotype has received attention in recent years as it is increasing in disease and is not included in current vaccines, while also having been reported to be associated with pneumococcal meningitis, the most severe form of IPD [51, 58]. Interestingly, in France and Spain, the expansion of 24F was mainly attributed to GPSC10, associated with penicillin and erythromycin resistance. Conversely, GPSC6, was not associated with penicillin resistance and was significantly associated with carriage. This lineage was most common among 24F isolates in Denmark, where antibiotic consumption is lower and more similar to Norway [51]. Further supporting the link between antibiotic consumption and the selection for specific GPSC lineages, the expansion of 24F in Japan, a country with a high consumption of erythromycin combined with low penicillin consumption, was driven by GPSC106, which exhibits erythromycin, but not penicillin resistance [51, 59]. The GPSC and susceptibility profiles of the limited number of 24F isolates observed in the current study suggest that the 24F epidemiology in Norway mirrors the observations from Denmark.

## Limitations

Here, we look at the major lineages in IPD across all age groups spanning 4 decades and the subsequent expansion of lineages with PCV13 non-vaccine types post-PCV13. While the majority of the IPD burden falls in the > 60 years age group, PCV13 is mainly given in the Norwegian childhood immunization program, though PCVs can be given in combination with PPV23 in the ≥ 65 age group when PPV23 is deemed insufficient protection by the general practitioner. However, it is likely the use of PCV13 in children that is driving these changes in adult disease. Estimating incidence from data that included all isolates with MICs > 2 μg/ml could have influenced our estimated IRRs for expanding NVT-lineages; however, MICs > 2 μg/ml were predominantly (53/62 85%) found in VTs and were extremely rare in the NVT expansions, only being detected in GPSC6-NVTs. Nonetheless, the presence of NVT isolates in GPSC6 with MICs of > 2 μg/ml is a concern. We estimated the incidence of each lineage by extrapolating from their prevalence in the sequenced isolates. This relies on deep sampling to limit noise but could have been affected by unknown and unintentional sampling bias despite the randomized sampling. The combined incidence estimates for each clone were equivalent to the known total IPD incidence.

## Conclusions

Our results suggest that selection for penicillin resistance has not been a substantial driver of the pneumococcal population dynamics in Norway. The multivalent conjugate vaccines PCV7 and PCV13 have, on the other hand, been major shapers of the Norwegian pneumococcal invasive disease population. This shaping has affected not only serotype distributions but also the underlying lineage dynamics. Capsule types are far from randomly distributed across GPSCs, and the most obvious effect of PCVs was to substantially reduce the incidence of GPSCs dominated by vaccine type capsules such as GPSCs 18 and 39, the two major serotype 14 lineages, in parallel with the expansion of GPSCs carrying capsules not covered by the PCVs, including GPSC19-22F. Furthermore, we show that selection for reduced penicillin susceptibility has not been a major shaper of serotype selection.

Of particular interest, two GPSCs, GPSC6 and GPCS7, were initially dominated by vaccine types and largely escaped the diminutive effect of vaccination through the expansion of PCV13 non-vaccine serotypes. Our results demonstrate that the majority of these serotype-GPSC combinations were circulating at low frequencies prior to the introduction of the vaccines and that these expanding lineages include multiple serotypes not targeted by any licensed pneumococcal pediatric vaccines (15A, 23A, 23B, and 24F). The expansion of particularly invasive PCV13-NVTs such as serotypes 8 (PPV23) and 22F (PPV23, PCV15, PCV20) and serotype 24F (PCV21-Merck V116) may also require greater PCV coverage in the over-65 age group including PCV + PPV combinations to increase effectiveness.

## Supplementary Information

Additional file 1: Fig S1. Root-to-tip analyses using BactDating on Gubbins output. GPSC6 to the left, GPSC7 to the right. Figure S2: Age distribution of cases underlying the historic pneumococcal genome dataset. A) Age-distribution over time in the historic dataset. The times of introduction of PCV7 in 2006 and PCV13 in 2011 are annotated with vertical dotted lines. B) Age distribution with the largest GPSCs in Norway. Figure S3: Main GPSCs across penicillin MIC values. The meningitis (≥ 0.06 µg/ml) and non-meningitis (> 2 µg/ml) breakpoints are indicated by dotted vertical lines. Fig S4. Age distribution within the eight major GPSCs. The three GPSCs annotated with ‘expansion’ and ‘collapse’ were all completely dominated by serotypes covered by PCV7. Yet, GPSC12 (serotype 3) expanded, whereas GPSC18 and GPSC39 (both serotype 14) collapsed, following the introduction of PCV7 (see Fig. 4). The three age distributions were significantly different as assessed by the non-parametric Kruskal–Wallis test (*p* = 0.00074). Further post hoc analyses using Dunn´s test with Bonferroni correction showed that the age distribution of GPSC39 was significantly different from the two other GPSCs, but found no significant difference between GPSC12 and GPSC18. Table S1. Estimated incidence rate ratios, pre-PCV7 versus post-PCV13, per GPSC.

Additional file 2. Strain metadata.

## Data Availability

Raw sequencing reads are available at ENA under study accession PRJEB77077. Sequence assemblies can be accessed and downloaded from from the GPS database Monocle data viewer [22] by filtering for Country = Norway. Isolate-level metadata are available in Additional file 2.

## References

[1] Winje BA, Vestrheim DF, White RA, Steens A. The risk of invasive pneumococcal disease differs between risk groups in Norway following widespread use of the 13-valent pneumococcal vaccine in children. Microorganisms. 2021;9. 10.3390/microorganisms9081774. 10.3390/microorganisms9081774 PMC8398338 34442853

[2] Løvlie A, Vestrheim DF, Aaberge IS, Steens A. Changes in pneumococcal carriage prevalence and factors associated with carriage in Norwegian children, four years after introduction of PCV13. BMC Infect Dis. 2020;20:29. 31924177 10.1186/s12879-019-4754-0 PMC6954625

[3] Vaxneuvance pneumococcal polysaccharide conjugate vaccine, authorisation note. Available from: https://www.ema.europa.eu/en/medicines/human/EPAR/vaxneuvance. Cited 2024 Apr 17.

[4] Prevenar 20 pneumococcal polysaccharide conjugate vaccine (20-valent, adsorbed), authorisation note. Available from: https://www.ema.europa.eu/en/medicines/human/EPAR/prevenar-20-previously-apexxnar. Cited 2024 Apr 17.

[5] Kapatai G, Sheppard CL, Al-Shahib A, Litt DJ, Underwood AP, Harrison TG, et al. Whole genome sequencing of Streptococcus pneumoniae: development, evaluation and verification of targets for serogroup and serotype prediction using an automated pipeline. PeerJ. 2016;4: e2477. 27672516 10.7717/peerj.2477 PMC5028725

[6] Bentley SD, Aanensen DM, Mavroidi A, Saunders D, Rabbinowitsch E, Collins M, et al. Genetic analysis of the capsular biosynthetic locus from all 90 pneumococcal serotypes. PLoS Genet. 2006;2. Available from: https://pubmed.ncbi.nlm.nih.gov/16532061/. Cited 2024 Mar 3. 10.1371/journal.pgen.0020031 PMC1391919 16532061

[7] Croucher NJ, Kagedan L, Thompson CM, Parkhill J, Bentley SD, Finkelstein JA, et al. Selective and genetic constraints on pneumococcal serotype switching. PLoS Genet. 2015;11: e1005095. 25826208 10.1371/journal.pgen.1005095 PMC4380333

[8] Bradshaw JL, Rafiqullah IM, Robinson DA, McDaniel LS. Transformation of nonencapsulated Streptococcus pneumoniae during systemic infection. Sci Rep. 2020;10:18932. 33144660 10.1038/s41598-020-75988-5 PMC7641166

[9] Croucher NJ, Hanage WP, Harris SR, McGee L, van der Linden M, de Lencastre H, et al. Variable recombination dynamics during the emergence, transmission and “disarming” of a multidrug-resistant pneumococcal clone. BMC Biol. 2014;12:49. 24957517 10.1186/1741-7007-12-49 PMC4094930

[10] Weinberger DM, Malley R, Lipsitch M. Serotype replacement in disease after pneumococcal vaccination. Lancet. 2011;378:1962–73. 21492929 10.1016/S0140-6736(10)62225-8 PMC3256741

[11] Lo SW, Gladstone RA, van Tonder AJ, Lees JA, du Plessis M, Benisty R, et al. Pneumococcal lineages associated with serotype replacement and antibiotic resistance in childhood invasive pneumococcal disease in the post-PCV13 era: an international whole-genome sequencing study. Lancet Infect Dis. 2019;19:759–69. 31196809 10.1016/S1473-3099(19)30297-X PMC7641901

[12] Luck JN, Tettelin H, Orihuela CJ. Sugar-coated killer: serotype 3 pneumococcal disease. Front Cell Infect Microbiol. 2020;10: 613287. 33425786 10.3389/fcimb.2020.613287 PMC7786310

[13] Park IH, Pritchard DG, Cartee R, Brandao A, Brandileone MCC, Nahm MH. Discovery of a new capsular serotype (6C) within serogroup 6 of Streptococcus pneumoniae. J Clin Microbiol. 2007;45:1225–33. 17267625 10.1128/JCM.02199-06 PMC1865839

[14] Feemster K, Weaver J, Buchwald U, Banniettis N, Cox KS, McIntosh ED, et al. Pneumococcal vaccine breakthrough and failure in infants and children: a narrative review. Vaccines (Basel). 2023;11. 10.3390/vaccines11121750. 10.3390/vaccines11121750 PMC10747311 38140155

[15] Corander J, Fraser C, Gutmann MU, Arnold B, Hanage WP, Bentley SD, et al. Frequency-dependent selection in vaccine-associated pneumococcal population dynamics. Nat Ecol Evol. 2017;1:1950–60. 29038424 10.1038/s41559-017-0337-x PMC5708525

[16] Gladstone RA, Devine V, Jones J, Cleary D, Jefferies JM, Bentley SD, et al. Pre-vaccine serotype composition within a lineage signposts its serotype replacement - a carriage study over 7 years following pneumococcal conjugate vaccine use in the UK. Microb Genom. 2017;3: e000119. 29026652 10.1099/mgen.0.000119 PMC5628697

[17] Gladstone RA, Lo SW, Lees JA, Croucher NJ, van Tonder AJ, Corander J, et al. International genomic definition of pneumococcal lineages, to contextualise disease, antibiotic resistance and vaccine impact. EBioMedicine. 2019;43:338–46. 31003929 10.1016/j.ebiom.2019.04.021 PMC6557916

[18] Li Y, Metcalf BJ, Chochua S, Li Z, Gertz RE Jr, Walker H, et al. Validation of β-lactam minimum inhibitory concentration predictions for pneumococcal isolates with newly encountered penicillin binding protein (PBP) sequences. BMC Genomics. 2017;18:621. 28810827 10.1186/s12864-017-4017-7 PMC5558719

[19] Epping L, van Tonder AJ, Gladstone RA, The Global Pneumococcal Sequencing Consortium, Bentley SD, Page AJ, et al. SeroBA: rapid high-throughput serotyping of Streptococcus pneumoniae from whole genome sequence data. Microb Genom. 2018;4. 10.1099/mgen.0.000186. 10.1099/mgen.0.000186 PMC6113868 29870330

[20] The Global Pneumococcal Sequencing Project. Available from: https://www.pneumogen.net/gps/.

[21] Pathogenwatch. A global platform for genomic surveillance. Available from: https://www.pathogen.watch.

[22] Monocle data viewer. Available from: https://data-viewer.monocle.sanger.ac.uk/project/gps.

[23] Bolger AM, Lohse M, Usadel B. Trimmomatic: a flexible trimmer for Illumina sequence data. Bioinformatics. 2014;30:2114–20. 24695404 10.1093/bioinformatics/btu170 PMC4103590

[24] Bankevich A, Nurk S, Antipov D, Gurevich AA, Dvorkin M, Kulikov AS, et al. SPAdes: a new genome assembly algorithm and its applications to single-cell sequencing. J Comput Biol. 2012;19:455–77. 22506599 10.1089/cmb.2012.0021 PMC3342519

[25] Lees JA, Harris SR, Tonkin-Hill G, Gladstone RA, Lo SW, Weiser JN, et al. Fast and flexible bacterial genomic epidemiology with PopPUNK. Genome Res. 2019;29:304–16. 30679308 10.1101/gr.241455.118 PMC6360808

[26] GPS tools. Available from: https://www.pneumogen.net.

[27] Wickham H, François R, Henry L, Müller K, Vaughan D. dplyr: a grammar of data manipulation. 2023. Available from: https://dplyr.tidyverse.org.

[28] Wickham H. ggplot2: Elegant Graphics for Data Analysis. New York: Springer-Verlag; 2016. See: https://ggplot2.tidyverse.org/authors.html#citation.

[29] Wickham H, Averick M, Bryan J, Chang W, McGowan LD, François R, et al. Welcome to the tidyverse . J Open Source Softw. 2019:1686. 10.21105/joss.01686.

[30] Neuwirth E. RColorBrewer: ColorBrewer Palettes. R package version 1.1-3. 2022. Available from: 10.32614/cran.package.rcolorbrewer.

[31] Schwengers O, Jelonek L, Dieckmann MA, Beyvers S, Blom J, Goesmann A. Bakta: rapid and standardized annotation of bacterial genomes via alignment-free sequence identification. Microb Genom. 2021;7. 10.1099/mgen.0.000685. 10.1099/mgen.0.000685 PMC8743544 34739369

[32] Seeman T. Snippy. Available from: https://github.com/tseemann/snippy.

[33] Croucher NJ, Page AJ, Connor TR, Delaney AJ, Keane JA, Bentley SD, et al. Rapid phylogenetic analysis of large samples of recombinant bacterial whole genome sequences using Gubbins. Nucleic Acids Res. 2015;43: e15. 25414349 10.1093/nar/gku1196 PMC4330336

[34] Minh BQ, Schmidt HA, Chernomor O, Schrempf D, Woodhams MD, von Haeseler A, et al. IQ-TREE 2: new models and efficient methods for phylogenetic inference in the genomic era. Mol Biol Evol. 2020;37:1530–4. 32011700 10.1093/molbev/msaa015 PMC7182206

[35] Didelot X, Croucher NJ, Bentley SD, Harris SR, Wilson DJ. Bayesian inference of ancestral dates on bacterial phylogenetic trees. Nucleic Acids Res. 2018;46: e134. 30184106 10.1093/nar/gky783 PMC6294524

[36] Drummond AJ, Suchard MA, Xie Dong, Rambaut A. Bayesian phylogenetics with BEAUti and the BEAST 1.7. Mol Biol Evol. 2012;29:1969–73. 10.1093/molbev/mss075. 10.1093/molbev/mss075 PMC3408070 22367748

[37] Hadfield J, Croucher NJ, Goater RJ, Abudahab K, Aanensen DM, Harris SR. Phandango: an interactive viewer for bacterial population genomics. Bioinformatics. 2018;34:292–3. 29028899 10.1093/bioinformatics/btx610 PMC5860215

[38] The Norwegian Surveillance System for Communicable Diseases. MSIS-statistikk. Available from: https://www.msis.no/.

[39] Vestrheim DF, Høiby EA, Aaberge IS, Caugant DA. Impact of a pneumococcal conjugate vaccination program on carriage among children in Norway. Clin Vaccine Immunol. 2010;17:325–34. 20107006 10.1128/CVI.00435-09 PMC2837970

[40] Savulescu C, Krizova P, Valentiner-Branth P, Ladhani S, Rinta-Kokko H, Levy C, et al. Effectiveness of 10 and 13-valent pneumococcal conjugate vaccines against invasive pneumococcal disease in European children: SpIDnet observational multicentre study. Vaccine. 2022;40:3963–74. 35637067 10.1016/j.vaccine.2022.05.011

[41] Groves N, Sheppard CL, Litt D, Rose S, Silva A, Njoku N, et al. Evolution of Streptococcus pneumoniae serotype 3 in England and Wales: a major vaccine evader. Genes. 2019;10. 10.3390/genes10110845. 10.3390/genes10110845 PMC6896183 31731573

[42] Palmborg A, Skovdal M, Molden T, Åhman H, Chen L, Banefelt J. Invasive pneumococcal disease among the elderly in the later era of paediatric pneumococcal conjugate vaccination-a longitudinal study over 10 years based on public surveillance data in the Nordics. PLoS ONE. 2023;18: e0287378. 37363884 10.1371/journal.pone.0287378 PMC10292715

[43] Swarthout TD, Henrion MYR, Thindwa D, Meiring JE, Mbewe M, Kalizang’Oma A, et al. Waning of antibody levels induced by a 13-valent pneumococcal conjugate vaccine, using a 3 + 0 schedule, within the first year of life among children younger than 5 years in Blantyre, Malawi: an observational, population-level, serosurveillance study. Lancet Infect Dis. 2022;22:1737–47. 36029796 10.1016/S1473-3099(22)00438-8 PMC10555849

[44] Swarthout TD, Fronterre C, Lourenço J, Obolski U, Gori A, Bar-Zeev N, et al. High residual carriage of vaccine-serotype Streptococcus pneumoniae after introduction of pneumococcal conjugate vaccine in Malawi. Nat Commun. 2020;11:2222. 32376860 10.1038/s41467-020-15786-9 PMC7203201

[45] Hanquet G, Krizova P, Dalby T, Ladhani SN, Nuorti JP, Danis K, et al. Serotype replacement after introduction of 10-valent and 13-valent pneumococcal conjugate vaccines in 10 countries, Europe. Emerg Infect Dis. 2022;28. Available from: https://pubmed.ncbi.nlm.nih.gov/34932457/. Cited 2024 Feb 27. 10.3201/eid2801.210734 PMC8714201 34932457

[46] Harboe ZB, Dalby T, Weinberger DM, Benfield T, Mølbak K, Slotved HC, et al. Impact of 13-valent pneumococcal conjugate vaccination in invasive pneumococcal disease incidence and mortality. Clin Infect Dis. 2014;59. Available from: https://pubmed.ncbi.nlm.nih.gov/25034421/. Cited 2024 Mar 3. 10.1093/cid/ciu524 25034421

[47] Croucher NJ, Harris SR, Fraser C, Quail MA, Burton J, van der Linden M, et al. Rapid pneumococcal evolution in response to clinical interventions. Science. 2011;331:430–4. 21273480 10.1126/science.1198545 PMC3648787

[48] Paton JC, Trappetti C. Streptococcus pneumoniae capsular polysaccharide. Microbiol Spectr. 2019;7. 10.1128/microbiolspec.GPP3-0019-2018. 10.1128/microbiolspec.gpp3-0019-2018 PMC11590643 30977464

[49] van Tonder AJ, Bray JE, Quirk SJ, Haraldsson G, Jolley KA, Maiden MCJ, et al. Putatively novel serotypes and the potential for reduced vaccine effectiveness: capsular locus diversity revealed among 5405 pneumococcal genomes. Microbial Genomics. 2016;2: e000090. 28133541 10.1099/mgen.0.000090 PMC5266551

[50] Gladstone RA, Lo SW, Goater R, Yeats C, Taylor B, Hadfield J, et al. Visualizing variation within Global Pneumococcal Sequence Clusters (GPSCs) and country population snapshots to contextualize pneumococcal isolates. Microb Genom. 2020;6. 10.1099/mgen.0.000357. 10.1099/mgen.0.000357 PMC7371119 32375991

[51] Lo SW, Mellor K, Cohen R, Alonso AR, Belman S, Kumar N, et al. Emergence of a multidrug-resistant and virulent Streptococcus pneumoniae lineage mediates serotype replacement after PCV13: an international whole-genome sequencing study. Lancet Microbe. 2022;3:e735–43. 35985351 10.1016/S2666-5247(22)00158-6 PMC9519462

[52] Belman S, Pesonen H, Croucher NJ, Bentley SD, Corander J. Estimating between country migration in pneumococcal populations. G3. 2024. 10.1093/g3journal/jkae058. 38507601 10.1093/g3journal/jkae058 PMC11152062

[53] Belman S, Lefrancq N, Nzenze S, Downs S, du Plessis M, Lo S, et al. Geographic migration and vaccine-induced fitness changes of Streptococcus pneumoniae. bioRxiv. 2023. 10.1101/2023.01.18.524577.

[54] Azarian T, Mitchell PK, Georgieva M, Thompson CM, Ghouila A, Pollard AJ, et al. Global emergence and population dynamics of divergent serotype 3 CC180 pneumococci. PLoS Pathog. 2018;14: e1007438. 30475919 10.1371/journal.ppat.1007438 PMC6283594

[55] Bertran M, D’Aeth JC, Abdullahi F, Eletu S, Andrews NJ, Ramsay ME, et al. Invasive pneumococcal disease 3 years after introduction of a reduced 1 + 1 infant 13-valent pneumococcal conjugate vaccine immunisation schedule in England: a prospective national observational surveillance study. Lancet Infect Dis. 2024;24:546–56. 38310905 10.1016/S1473-3099(23)00706-5

[56] Løchen A, Truscott JE, Croucher NJ. Analysing pneumococcal invasiveness using Bayesian models of pathogen progression rates. bioRxiv. 2021. p. 2021.09.01.458483. Available from: https://www.biorxiv.org/content/10.1101/2021.09.01.458483v1. Cited 2021 Oct 26. 10.1371/journal.pcbi.1009389 PMC8901055 35176026

[57] Yamba Yamba L, Uddén F, Fuursted K, Ahl J, Slotved H-C, Riesbeck K. Extensive/multidrug-resistant pneumococci detected in clinical respiratory tract samples in Southern Sweden are closely related to international multidrug-resistant lineages. Front Cell Infect Microbiol. 2022;12: 824449. 35392607 10.3389/fcimb.2022.824449 PMC8981583

[58] Ouldali N, Levy C, Varon E, Bonacorsi S, Béchet S, Cohen R, et al. Incidence of paediatric pneumococcal meningitis and emergence of new serotypes: a time-series analysis of a 16-year French national survey. Lancet Infect Dis. 2018;18. Available from: https://pubmed.ncbi.nlm.nih.gov/30049623/. Cited 2024 Mar 8. 10.1016/S1473-3099(18)30349-9 30049623

[59] Ubukata K, Takata M, Morozumi M, Chiba N, Wajima T, Hanada S, et al. Effects of pneumococcal conjugate vaccine on genotypic penicillin resistance and serotype changes, Japan, 2010–2017. Emerg Infect Dis. 2018;24:2010–20. 30334707 10.3201/eid2411.180326 PMC6200004

