# Supplementary material for: A genome-based survey of invasive pneumococci in Norway over four decades reveals lineage-specific responses to vaccination
Source: Genome Med. 2024 Oct 25;16:123. doi: 10.1186/s13073-024-01396-3 (PMC11515192; doi:10.1186/s13073-024-01396-3)
Supplement: Supplementary file 1 — Additional file 1: Fig S1. Root-to-tip analyses using BactDating on Gubbins output. GPSC6 to the left, GPSC7 to the right. Figure S2: Age distribution of cases underlying the historic pneumococcal genome dataset. A) Age-distribution over time in the historic dataset. The times of introduction of PCV7 in 2006 and PCV13 in 2011 are annotated with vertical dotted lines. B) Age distribution with the largest GPSCs in Norway. Figure S3: Main GPSCs across penicillin MIC values. The meningitis (≥ 0.06 µg/ml) and non-meningitis (> 2 µg/ml) breakpoints are indicated by dotted vertical lines. Fig S4. Age distribution within the eight major GPSCs. The three GPSCs annotated with ‘expansion’ and ‘collapse’ were all completely dominated by serotypes covered by PCV7. Yet, GPSC12 (serotype 3) expanded, whereas GPSC18 and GPSC39 (both serotype 14) collapsed, following the introduction of PCV7 (see Fig. 4). The three age distributions were significantly different as assessed by the non-parametric Kruskal–Wallis test (p = 0.00074). Further post hoc analyses using Dunn´s test with Bonferroni correction showed that the age distribution of GPSC39 was significantly different from the two other GPSCs, but found no significant difference between GPSC12 and GPSC18. Table S1. Estimated incidence rate ratios, pre-PCV7 versus post-PCV13, per GPSC. [file 13073_2024_1396_MOESM1_ESM.pdf]

Additional file 1

Supplementary figures and data

A genome-based survey of invasive pneumococci in Norway over four decades reveals lineage-specific responses to vaccination

Eldholm V, Osnes MN, Bjørnstad ML, Straume D, Gladstone RA

Contents

Figures S1-S4
Table S1

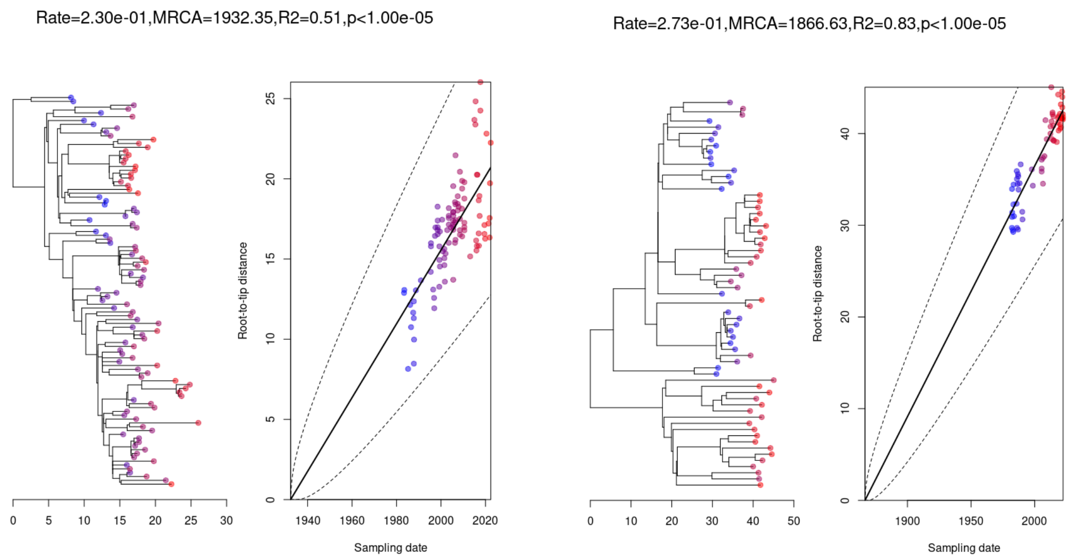

Fig S1. Root-to-tip analyses using BactDating on Gubbins output. GPSC6 to the left, GPSC7 to the right.

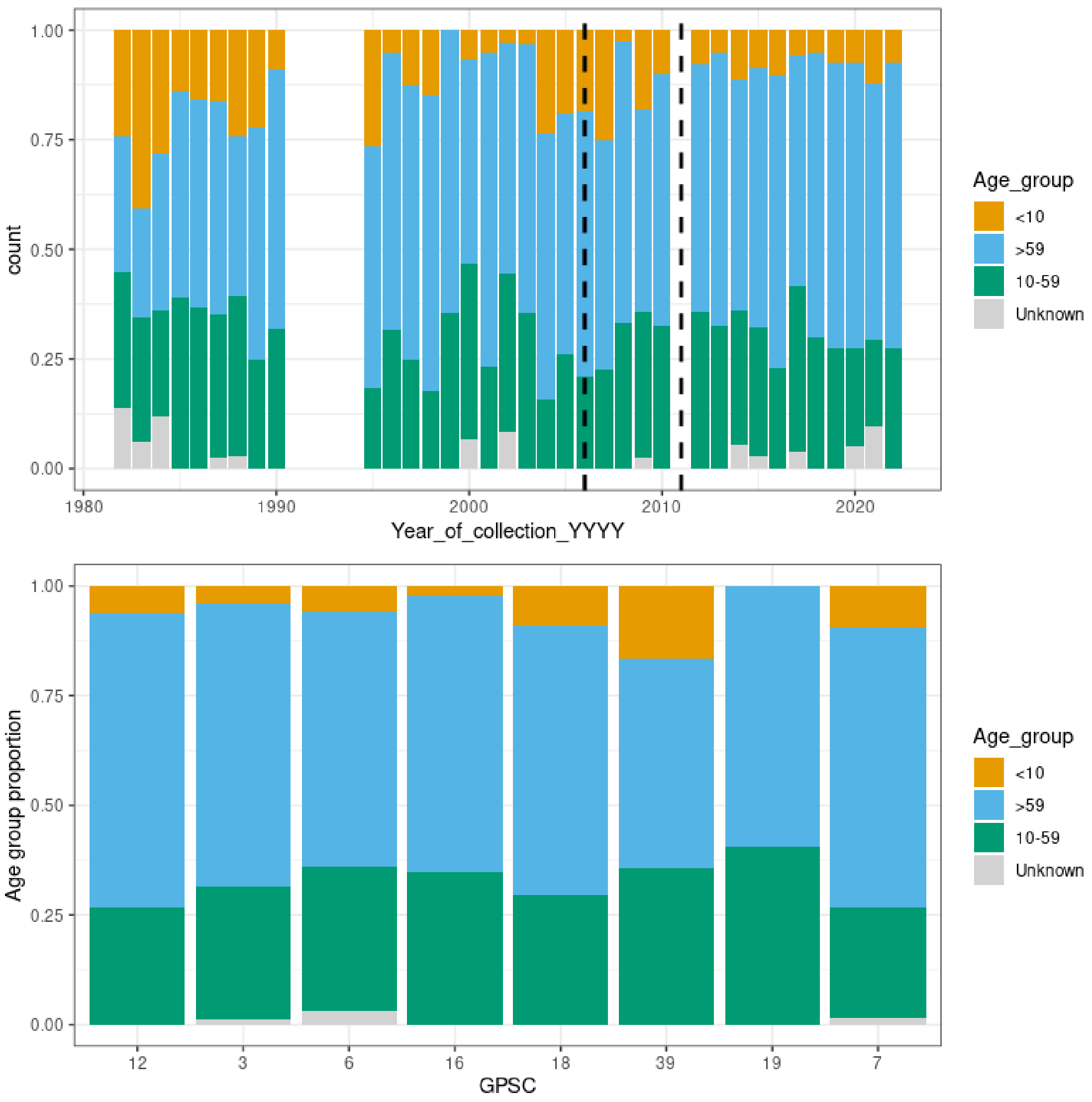

Figure S2: Age distribution of cases underlying the historic pneumococcal genome dataset. **A)** Age-distribution over time in the historic dataset. The times of introduction of PCV7 in 2006 and PCV13 in 2011 are annotated with vertical dotted lines. **B)** Age distribution with the largest GPSCs in Norway.

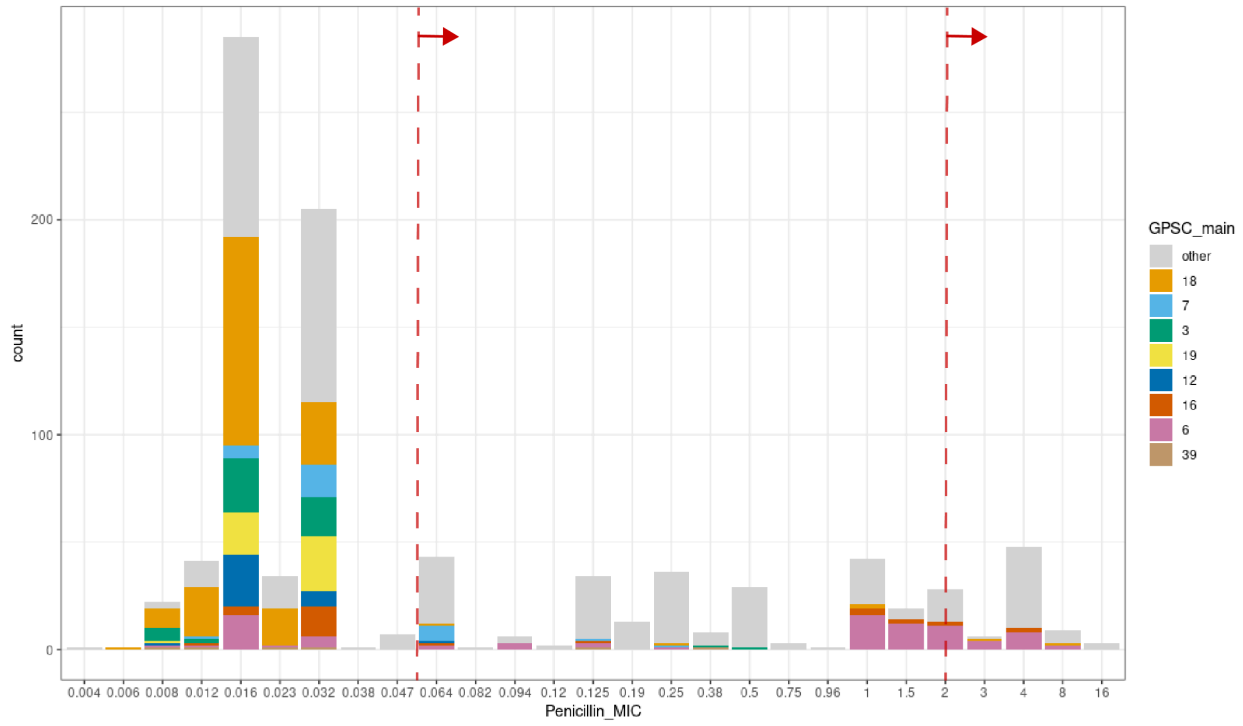

Figure S3: Main GPSCs across penicillin MIC values. The meningitis (≥ 0.06 µg/ml) and non-meningitis (> 2 µg/ml) breakpoints are indicated by dotted vertical lines.

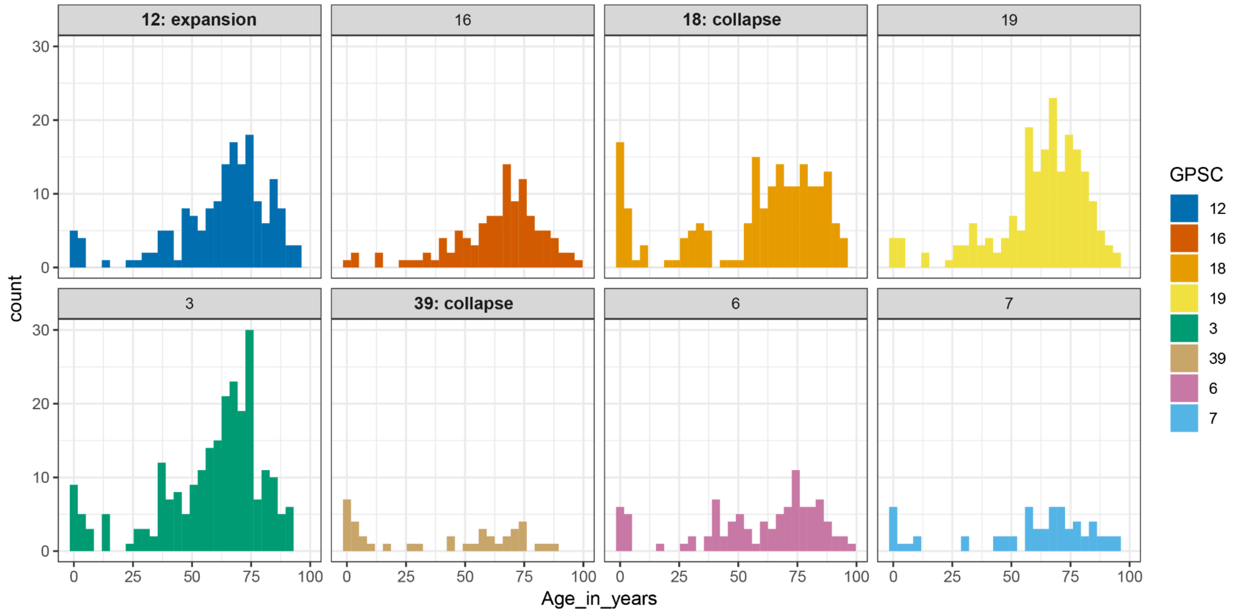

Fig S4. Age distribution within the eight major GPSCs. The three GPSCs annotated with ‘expansion’ and ‘collapse’ were all completely dominated by serotypes covered by PCV7. Yet, GPSC12 (serotype 3) expanded, whereas GPSC18 and GPSC39 (both serotype 14) collapsed,

following the introduction of PCV7 (see Fig. 4). The three age distributions were significantly different as assessed by the non-parametric Kruskal-Wallis test (p=0.00074). Further post hoc analyses using Dunn´s test with Bonferroni correction showed that the age distribution of GPSC39 was significantly different from the two other GPSCs, but found no significant difference between GPSC12 and GPSC18.

Table S1. Estimated incidence rate ratios, pre-PCV7 versus post-PCV13, per GPSC

GPSC	ST	IRR VT	IRR NVT	PCV13 NVTs count post PCV13	PCV13-NVTs (% of NVTs)	PCV15 NVT coverage	PCV20 NVT coverage	PCV21 NVT coverage	PPV23 NVT coverage	NVT penicillin resistance (meningitis, non-meningitis#)
**3**	53	NA	3.6 [1.4-9.6]*	213	8 (70%), 33F (15%), 11A (10%), 11E (3%)	16%	97%	11%	97%	4%, 0%
**6**	162	0.05 [0.02-0.2]**	19.1 [9.7-39.2]**	54	15A (28%), 11A (7%), 23A (9%), 15C (2%)	0%	7%	100%	7%	9%, 0%
**7**	42	0.01 [0.001-0.1]*	23.3 [6.2-103.0]**	99	23A (62%), 23B (38%)	0%	0%	100%	0%	18%, 0%
**12**	180	9.6 [3.5-28.9]*	NA	1	35B (100%)	0%	0%	100%	0%	0%, 0%
**16**	66	0.5 [0.1-1.9]	2.1 [0.8-6.0]	81	9N (100%)	0%	0%	0%	100%	4%, 0%
**18**	9	0.01 [0.002-0.06]**	NA	3	7C (100%)	0%	0%	0%	0%	0%, 0%
**19**	433	NA	22.0 [7.3-74.0]**	188	22F (100%)	100%	100%	0%	100%	0%, 0%
**39**	124	0.04 [0.02-0.1]**	NA	0	None	NA	NA	NA	NA	0%, 0%
9	63	NA	9.8 [1.8-67.9]*	28	15A (96%), 15B (4%)	0%	4%	96%	4%	100%, 0%
29	1692	NA	3.5 [1.7-7.5]*	8	6C (100%)	0%	0%	0%	0%	0%, 0%
35	1551	NA	15.8 [1.9-195.3]*	23	10A (96%), 6C (4%)	0%	96%	0%	96%	7%, 0%
36	446	NA	5.7 [1.7-22.2]*	76	35F (71%), 10A (28%), 11A (1%)	0%	29%	1%	29%	8%,0%
46	30	NA	23.5 [4.2-168.3]*	21	16F (100%)	0%	0%	100%	0%	0%, 0%
47	386	NA	2.5 [1.4-4.4]*	6	6C (100%)	0%	0%	0%	0%	100%, 0%
72	198	NA	5.7 [2.8-11.8]**	17	35B (94%), 35D (6%)	0%	0%	94%	0%	30%, 0%
89	1379	NA	4.0 [1.9-8.7]*	8	6C (100%)	0%	0%	0%	0%	0%, 0%
139	6524	NA	2.6 [1.5-4.6]*	7	10B (86%), 33B (14%)	0%	0%	0%	0%	0%, 0%

The top eight GPSCs are presented in bold, all remaining significantly increasing GPSC-NVTs are additionally included. Sequence type (ST). *p<0.05, **p<0.0001
#As all > 2 µg/ml MIC IPD isolates were sequenced but are otherwise rare these were excluded from the percentage resistance to prevent an inflation of the proportion that were resistant. The only GPSC among the 17 lineages presented in this table with isolates exhibiting MIC >=4 µg/ml, was GPSC6, n=6 were excluded on this basis. Incidence was estimated for the IRRs by extrapolating from the prevalence in sequenced isolates to the known total IPD cases.
